# Geomagnetic disturbance associated with increased vagrancy in migratory landbirds

**DOI:** 10.1038/s41598-022-26586-0

**Published:** 2023-01-09

**Authors:** Benjamin A. Tonelli, Casey Youngflesh, Morgan W. Tingley

**Affiliations:** 1 Department of Ecology and Evolutionary Biology, University of California, Los Angeles, CA 90095 USA; 2 Present Address: Ecology, Evolution and Behavior Program, Michigan State University, East Lansing, Michigan 48824 USA

**Keywords:** Animal migration, Ecology

## Abstract

Rare birds known as “accidentals” or “vagrants” have long captivated birdwatchers and puzzled biologists, but the drivers of these rare occurrences remain elusive. Errors in orientation or navigation are considered one potential driver: migratory birds use the Earth’s magnetic field—sensed using specialized magnetoreceptor structures—to traverse long distances over often unfamiliar terrain. Disruption to these magnetoreceptors or to the magnetic field itself could potentially cause errors leading to vagrancy. Using data from 2 million captures of 152 landbird species in North America over 60 years, we demonstrate a strong association between disruption to the Earth’s magnetic field and avian vagrancy during fall migration. Furthermore, we find that increased solar activity—a disruptor of the avian magnetoreceptor—generally counteracts this effect, potentially mitigating misorientation by disabling the ability for birds to use the magnetic field to orient. Our results link a hypothesized cause of misorientation to the phenomenon of avian vagrancy, further demonstrating the importance of magnetoreception among the orientation mechanisms of migratory birds. Geomagnetic disturbance may have important downstream ecological consequences, as vagrants may experience increased mortality rates or facilitate range expansions of avian populations and the organisms they disperse.

## Introduction

Rare events are increasingly being recognized as important forces in structuring ecological systems^1–5^. Such events, like the anomalous long-distance dispersal of an organism or the breakthrough introduction of a novel pathogen can have profound impacts, such as rapid range expansion or severe population decline^6–8^. One of the best-documented rare ecological events is the appearance of individual birds well outside their expected range. The unlikely appearance of these individuals, called “vagrants” or “accidentals,” has long motivated scientific thought and elicited wonder among both professional and amateur ornithologists^9–12^. As a result, vagrant birds—such as a European Robin (*Erithacus rubecula*) in Beijing or a Steller’s Sea Eagle (*Haliaeetus pelagicus*) near Boston—can become ephemeral celebrities, attracting large crowds and bolstering local economies^13,14^. The phenomenon of vagrancy has been hypothesized to have downstream ecological ramifications, both positive and negative. While vagrants are thought to be unlikely to survive and reproduce^9^, those that do survive may also drive the range expansion of populations by seeding individuals into newly ecologically suitable areas^5,9,15^.

Many factors may contribute to the phenomenon of avian vagrancy, including weather^10,12,16,17^, population fluctuations^18,19^, and genetic abnormalities^20^, but one compelling theory behind vagrancy is that it can result from the failure of a bird’s navigation system^21,22^. While birds use a variety of information sources to orient themselves, including celestial, solar, and olfactory cues^23,24^, an important mechanism employed across species is magnetoreception^25^, whereby birds orient themselves using the Earth’s magnetic field^26–29^. In both laboratory and field studies, birds have been shown to make behavioral changes and orientation shifts based on manipulation of magnetic cues^30–35^. Birds placed in experimentally altered magnetic fields and exposed to radiofrequency noise either attempt to fly along incorrect headings (misorientation) or lose their sense of direction altogether (disorientation)^33,36,37^.

**Figure 1:**
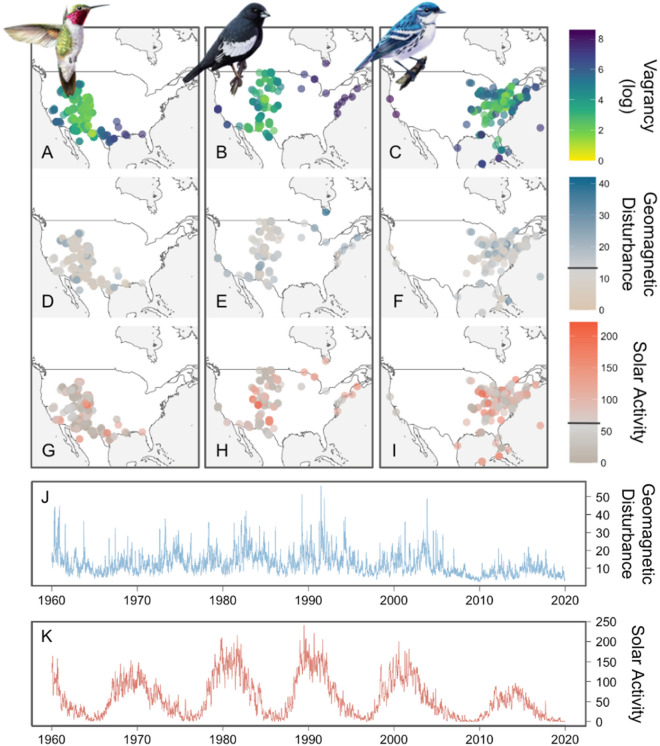
Species-specific banding data provides replicate tests for the causes of avian vagrancy. For each bird species (n_fall_ = 150, n_spring_ = 124) we calculated a vagrancy index for every banding record (Fig. S1), numerically representing the spatio-temporal rarity of the record (**A**–**C**). Each record was also matched by date to 21-day rolling indices of geomagnetic disturbance (**D**–**F**), and solar activity (**G**–**I**). Horizontal bars represent average geomagnetic disturbance and solar activity during the study period. Plots show data for three taxonomically representative species during fall migration: the Broad-tailed Hummingbird (*Selasphorus platycercus*; **A, D, G**), Lark Bunting (*Calamospiza melanocorys;* **B**, **E**, **H**), and Cerulean Warbler (*Setophaga cerulea;* **C**, **F**, **I**). 21-day rolling average for (**J**) geomagnetic disturbance (global Ap) and (**K**) solar activity (American relative sunspot number) over the study period (1960–2019). Maps were created using the packages rnaturalearth^47^(v. 0.1.0, https://github.com/ropensci/rnaturalearth) and ggplot2^48^(v. 3.3.5, https://ggplot2.tidyverse.org).

Disruption to magnetic orientation may also occur in nature. The Earth’s magnetic field varies over multiple time scales (Fig. 1), with disruptive shifts occurring rapidly during geomagnetic storms which disproportionately affect higher latitudes^38^. These geomagnetic storms have been shown to result in scattered orientation headings of nocturnally migrating birds^39^, the loss of domesticated pigeons during recreational races^40^, and, in one case, to have coincided with an otherwise inexplicable fallout of vagrants over the British Isles^12,41^. In addition, solar activity—which also varies widely over multiple time scales and generally precedes geomagnetic activity (Fig. 1)^42^—may interfere with migration by increasing the level of atmospheric radiofrequency noise, potentially disabling the avian magnetoreceptor and leading to disorientation^33^. Increased solar activity has been associated with negative consequences in multiple migratory species, including reduced recruitment in endangered cranes^43^ and increased stranding of whales^44,45^, although the exact mechanisms behind these phenomena are the subject of ongoing debate^46^. Consequently, while both disruption of the Earth’s magnetic field and changing solar activity have been hypothesized to affect the ability of migratory birds to navigate or orient, the existence, extent, or strength of these potential effects on avian migration is currently unknown.

Here, we investigate whether vagrancy in migratory landbirds is associated with disturbances in the Earth’s magnetic field and/or with increased solar activity. To investigate whether higher rates of vagrancy are associated with either phenomenon, we analyzed a dataset of fall and spring records of birds captured and affixed with uniquely numbered metal leg bands as part of the North American Bird Banding Program^49^. Our filtered dataset comprises 2,226,936 records of 152 migratory bird species (n_fall_ = 150, n_spring_ = 124) between 1960 and 2019. Rather than use a binary classification of vagrancy for individual records, we measured the spatiotemporal rarity of each record by comparing capture locations to the modeled expected range of each species on the date of banding (Fig. 1, S1). We then estimated the degree to which this continuous measure of spatiotemporal rarity—our vagrancy index—is associated with indices of either geomagnetic disturbance (global Ap index, Fig. 1) or solar activity (measured by proxy by the American relative sunspot number, Fig. 1) using a hierarchical Bayesian model. Our modeling approach used a random slopes and random intercepts structure that simultaneously estimated species-specific and group-level effects on vagrancy while quantifying uncertainty around these estimates. This random intercept structure accounted for interannual variation in vagrancy that might be due to alternative mechanisms, including weather, population fluctuations, and range shifts. As geomagnetic storms and solar activity are phenomenon that occur independently of these other likely drivers of interannual fluctuations in vagrancy, our inferential approach and statistical model provides the first large-scale test of the role of these factors on vagrancy while controlling for potential confounds.

## Results

**Figure 2:**
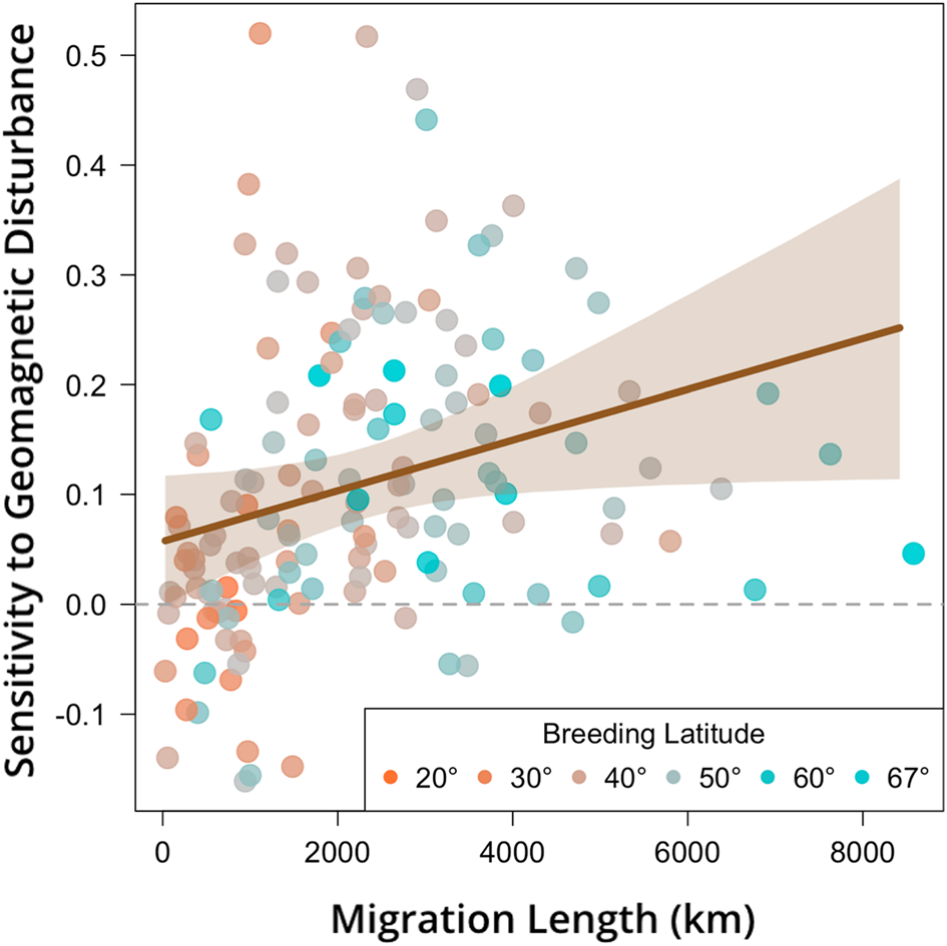
Species that migrate further show increased sensitivity of vagrancy to geomagnetic disturbance in the fall. Longer-distance migrants are more likely to rely on magnetoreception and thus are more susceptible to misorientation. There was no simultaneous effect of average breeding latitude (°N) on sensitivity to geomagnetic disturbance. Each point represents the model-derived posterior median estimate of sensitivity ($\mu_{{\beta_{i} }}$, Eq. 2,3) for an individual bird species (n = 150), while the brown line and envelope show the model-derived posterior median estimate and 95% CrI for the effect of migration length on sensitivity to geomagnetic disturbance.

We found, with high confidence, that vagrancy across North American landbirds is associated with increased geomagnetic disturbance in the fall migration season (posterior median $\omega_{fall,geom. dist.}$ = 0.109; 95% credible interval (CrI) = 0.077 to 0.142; proportion of posterior *p*(> 0) = 1; Table 1, Eq. 3), suggesting that orientation errors contribute to vagrancy in the majority of species in our dataset (83% of posterior median estimates of species-specific sensitivity, $\mu_{{\beta_{i,fall, geom. dist.} }}$ > 0; n = 150 species; Fig. 2 and S3, Eq. 3). For the typical species—defined by the mean species-level effect—an increase in geomagnetic disturbance of two standard deviations above average conditions is associated with a 24% increase in the median predicted vagrancy index of all records and a 250% increase in the number of spatiotemporally rare banding records (from 2% of all records at average geomagnetic disturbance to 5% under elevated conditions; Fig. S2).

**Table 1:** Parameter estimates (posterior median and 95% credible interval) from four hierarchical Bayesian models (columns) testing geomagnetic disturbance or solar activity (i.e., radiofrequency noise) on vagrancy during fall or spring migration seasons.

Potential driver of vagrancy	Geomagnetic disturbance		Solar activity	
Season	Fall	Spring	Fall	Spring
**Model parameter**				
Global effect of driver ($\omega$)	**0.109**	0.006	− 0.023	0.012
	**(0.077, 0.142)**	(− 0.017, 0.028)	(− 0.063, 0.017)	(− 0.037, 0.060)
	***p*(> 0) = 1**	*p*(> 0) = 0.70	*p*(< 0) = 0.86	*p*(> 0) = 0.68
Effect of age on vagrancy ($\lambda$)	**0.054**	**0.042**	**0.059**	**0.044**
	**(0.013, 0.098)**	**(0.019, 0.064)**	**(0.013, 0.103)**	**(0.022, 0.067)**
	***p*(> 0) = 0.99**	***p*(> 0) = 1**	***p*(> 0) = 0.99**	***p*(> 0) = 1**
Effect of age on sensitivity of vagrancy to driver ($\mu_{\nu }$)	0.006	− 0.008	− 0.015	0.005
	(− 0.011, 0.023)	(− 0.022, 0.018)	(− 0.032, 0.002)	(− 0.008, 0.019)
	*p*(> 0) = 0.76	*p*(< 0) = 0.88	*p*(< 0) = 0.95	*p*(> 0) = 0.76
Effect of migration length on species-specific sensitivity to driver ($\psi$)	**0.039**	− 0.007	0.005	− 0.044
	**(0.002, 0.075)**	(− 0.032, 0.018)	(− 0.040, 0.050)	(− 0.097, 0.011)
	***p*(> 0) = 0.98**	*p*(< 0) = 0.72	*p*(> 0) = 0.58	*p*(< 0) = 0.94
Effect of breeding latitude on species-specific sensitivity to driver ($\eta$)	− 0.004	0.005	− 0.046	**0.064**
	(− 0.041, 0.034)	(− 0.021, 0.032)	(− 0.094, 0.001)	**(0.008, 0.120)**
	*p*(< 0) = 0.57	*p*(> 0) = 0.65	*p*(< 0) = 0.97	***p*(> 0) = 0.99**

Proportion of the posterior greater than 0 for positive medians *p*(> 0), or less than 0 for negative medians *p*(< 0), are also reported. Parameters in bold indicate that there is strong posterior evidence (95% credible interval does not include zero) in support of a hypothesized effect. Parameter symbols correspond to Eqs. (1–3).

In examining species-specific variation in the strength of the relationship between geomagnetic disturbance and vagrancy—defined here as a species’ sensitivity—we hypothesized that species that migrate further would be more sensitive to magnetic field disruptions. Long-distance migrants likely both rely more on magnetic cues when navigating during migration and make more consequential mistakes during successive, long-distance flights compared to those of short-distance migrants^50,51^. We also hypothesized that species that breed closer to the poles would show greater sensitivity to geomagnetic disturbance because they would be subject to the more extreme perturbations of the magnetic field present at high latitudes^52^. By modeling the effect of these species-specific traits on vagrancy sensitivity, we found that sensitivity during fall to geomagnetic disturbance was generally stronger among species that migrate further (posterior median $\psi_{fall,geom.dist.}$ = 0.039; 95% CrI = 0.002 to 0.075; *p*(> 0) = 0.98; Table 1, Fig. 2, Eq. 3), supporting our first hypothesis. However, we did not find evidence that breeding at higher latitudes—controlling for migration distance—was associated with greater sensitivity to geomagnetic disturbance (posterior median $\eta_{fall,geom.dist}$ = − 0.004; 95% CrI = − 0.041 to 0.034; *p*(< 0) = 0.57; Table 1, Eq. 3).

Young birds rely on innate cues that are likely inferior to learned ones, and juveniles are subsequently known to appear as vagrants at higher rates^16^. We estimated the effect of age on vagrancy rates and the extent to which juveniles respond more strongly to geomagnetic disturbance compared to adults. As predicted, we found higher baseline levels of fall vagrancy among hatch-year birds compared to adults (posterior median of hatch-year intercept offset, $\mu_{{\lambda_{fall,geom.dist} }}$ = 0.054; 95% CrI = 0.013 to 0.098; *p*(> 0) = 0.99; Table 1, Eq. 2). However, we did not find that hatch-year birds were more sensitive to geomagnetic disturbances than adults during fall migration (posterior median $\mu_{{\nu_{fall,geom.dist} }}$ = 0.006; 95% CrI = − 0.011 to 0.023; *p*(> 0) = 0.76; Table 1, Eq. 2). These findings suggest that although younger birds are more likely to be vagrants, this difference cannot be attributed to a differential sensitivity to geomagnetic disturbance.

In contrast to fall migration, we found only weak evidence for a relationship between vagrancy during spring migration and either geomagnetic disturbance (posterior median $\omega_{spring,geom. dist.}$ = 0.006; 95% CrI = − 0.017 to 0.028; *p*(> 0) = 0.70; Table 1, Eq. 3) or solar activity (posterior median $\omega_{spring,solar activity}$ = 0.012, 95% CrI = − 0.037 to 0.060; *p*(> 0) = 0.68; Table 1, Eq. 3), despite similarly strong geomagnetic disturbance (mean 21-day Ap index of all banding records during fall = 12.90, and spring = 13.32) and solar activity during the fall and spring (mean 21-day sunspot number of all banding records during fall = 56.84, and spring = 55.38). Sampling biases in the underlying banding data could obscure a springtime effect; the spatiotemporal sampling of northern hemisphere banding sites preferentially captures individuals on or nearing their breeding sites during the late-migratory period, thus not capturing birds during the initial migration period when magnetic orientation may be most important for orientation (Fig. S7)^50,51^. In a post-hoc analysis to test this sampling-based hypothesis, we found that the strongest effect of geomagnetic disturbance on spring vagrancy is among species that winter further north and are thus more likely to be captured near the start of spring migration (Fig. S8). Alternatively, although unlikely, our inability to find a strong, assemblage-wide association of vagrancy with geomagnetic disturbance during spring migration may also suggest that birds are more reliant on other orientation mechanisms at this time of year—such as solar or celestial cues—mitigating the possibility of errors due to faulty magnetic orientation.

**Figure 3:**
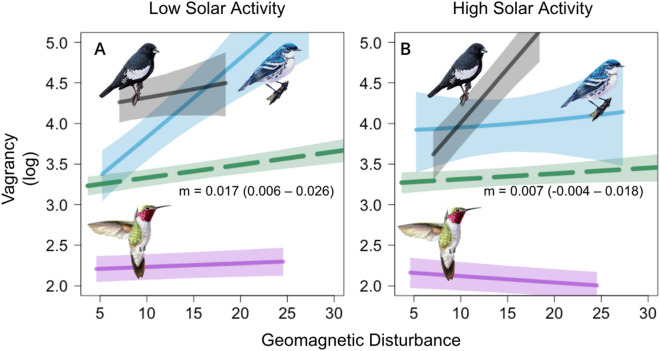
Effect of geomagnetic disturbance on vagrancy varies based on levels of solar activity. The effect of geomagnetic disturbance under low solar activity (**A**, 21-day sunspot number = 20, − 0.75 SD from mean) and high solar activity (**B**, 21-day sunspot number = 131, +1.5 SD from mean) conditions in the fall migration season across all species (green dotted line with slope and 95% CrI envelope) and three representative species: Broad-tailed Hummingbird (*Selasphorus platycercus,* magenta line), Lark Bunting (*Calamospiza melanocorys*, black line), and Cerulean Warbler (*Setophaga cerulea,* blue line), each with 95% CrI envelope. The effect of geomagnetic disturbance is mitigated under high solar activity conditions broadly (Table 2), but varies widely across species (Fig. S5).

Although our results strongly indicate that higher geomagnetic disturbance increases fall vagrancy rates, we found that sunspot number—a proxy for solar activity and radiofrequency noise—was potentially negatively associated with fall vagrancy (posterior median $\omega_{fall,solar activity}$ = − 0.023; 95% CrI = − 0.063 to 0.017; *p*(< 0) = 0.86; Table 1, Fig S4, Eq. 3), particularly among hatch-year birds ($\mu_{\nu ,fall,solar activity}$ = − 0.015; 95% CrI = − 0.032 to 0.002; *p*(< 0) = 0.95; Table 1, Eq. 3). This was contrary to our hypothesis that solar activity would cause disorientation and increase vagrancy, and was surprising given the positive correlation between solar activity and geomagnetic disturbance (21-day rolling average, Pearson’s correlation = 0.358, *p* < 0.001). Upon discovery of this negative association, we considered a potential interaction between solar activity and geomagnetic disturbance on avian vagrancy and tested for this interaction with a modified version of our statistical model using data from the same species sets for both spring and fall migration (see “Methods”, “Models 5–6: interactive effect of geomagnetic disturbance and solar activity on vagrancy in spring or fall” sections). Results from this interaction model provide strong evidence for a negative interaction during fall migration, suggesting that during periods of high solar activity, birds are less sensitive to the effect of geomagnetic disturbance (posterior median $\mu_{{\omega_{fall} }}$ = − 0.025; 95% CrI = − 0.047 to − 0.003; *p*(< 0) = 0.99; Table 1, Fig. 3, Eq. 5). The explanatory power of our fall interaction model varied across species, with a median of 5.9% of total variance in vagrancy explained by geomagnetic disturbance and solar activity (species-specific variance explained ranged from 0.3 to 30%; Supplement 2). A weaker and less certain effect was also observed during spring migration (posterior median $\mu_{{\omega_{spring} }}$ = − 0.0054; 95% CrI = − 0.022 to 0.011; *p*(< 0) = 0.74; Table 2, Eq. 5). Experimental studies have established that disruptive radiofrequency noise—like that present during periods of increased solar activity—can cause disorientation by disabling the ability for birds to orient using magnetic information^31^. Our results suggest that the combination of high solar activity and geomagnetic disturbance leads to either a pause in migration or a switch to other cues during fall migration—either would ultimately mitigate the misorientation effect of simultaneously high geomagnetic disturbance.

**Table 2:** Parameter estimates (posterior median and 95% credible interval) for the model testing for an interaction between geomagnetic disturbance and radiofrequency noise.

Season	Fall	Spring
**Model parameter**		
Effect of geomagnetic disturbance ($\mu_{\beta }$)	**0.079**	0.020
	**(0.051, 0.108)**	(− 0.005, 0.045)
	***p*(> 0) = 1**	*p*(> 0) = 0.94
Effect of solar activity ($\mu_{\theta }$)	− 0.020	0.004
	(− 0.049, 0.008)	(− 0.028, 0.037)
	*p*(< 0) = 0.91	*p*(> 0) = 0.59
Geomagnetic Disturbance x Solar Activity ($\mu_{\omega }$)	− **0.025**	− 0.005
	**(**− **0.047, **− **0.003)**	(− 0.022, 0.011)
	***p*(< 0) = 0.99**	*p*(< 0) = 0.74

Proportion of the posterior greater than 0 for positive medians *p*(> 0), or less than 0 for negative medians *p*(< 0), are also reported. Parameters in bold indicate that there is strong posterior evidence (95% credible interval does not include zero) in support of a hypothesized effect. Parameter symbols correspond to Eqs. (4–5).

Despite the generalized, assemblage-wide effect of solar activity across 150 landbird species, evaluation of species-specific relationships showed remarkably high variability (Fig. S5, S6). Specifically, while birds generally show decreased vagrancy under high solar activity in the fall (Table 2, Fig. S5), 20% of species experienced greater vagrancy under higher solar activity conditions (posterior median 95% CrI estimates $\theta_{{i_{fall} }}$ > 0, Fig. S5 Eq. 5). In particular, we found that species like the Lark Bunting (*Calamospiza melanocorys*), which migrate diurnally—a trait more common among raptors and soaring birds than songbirds and which describes 24% of species in our fall dataset—were more sensitive to solar activity than nocturnally migrating species (t-test, diff. between mean sensitivity,$\theta_{{i_{fall} }}$, in nocturnal, diurnal groups = 0.062, t = − 2.03, *p* = 0.047; Fig. S9. Because solar radiofrequency noise primarily impacts the sunlit side of the Earth^53^, diurnal migrants may be differentially affected by increased solar activity, a result that may explain the previously observed negative effect of solar activity on the successful migration of diurnally migrating cranes^43^. In sum, solar activity may act as a driver of vagrancy in some daytime migrants but mitigate vagrancy for nocturnally migrating species.

## Discussion

Taken together, our results provide macroecological evidence in support of the widespread use and importance of magnetoreception in migratory landbirds, and the common reliance of birds on favorable solar and magnetic conditions to migrate accurately. Our results identify a new mechanism contributing to the phenomenon of avian vagrancy across dozens of species, and adds support to a growing body of literature demonstrating the sensitivity of different animals—from whales to ants to birds—to geomagnetic disturbances and radiofrequency noise^45,46,54^.

Laboratory studies that further test the nuanced behavioral responses within species and across taxa are best suited to facilitate the unraveling of more complex interactions between phylogeny, age, migration timing, and sensitivity to geomagnetic disturbance and solar activity^31,37,55^. These tests could investigate, with greater granularity, our finding that geomagnetic disturbance leads to vagrancy among fall migrants and similarly affects juvenile and adult birds during this season. Understanding which and when certain populations are sensitive to geomagnetic disturbance and solar activity can inform ongoing debates on the biological structures responsible for magnetoreception^28^ and the primacy of magnetoreception among orientation methods^56,57^. Much is still unknown and heavily debated in the study of avian orientation and magnetoreception, but here we demonstrate one macroecological ramification—vagrancy—that can be used as both an inferential tool in this area of study and a further justification of its importance.

Geomagnetic disturbance and solar activity are just one set of multiple non-exclusive causes of vagrancy in birds. Our fall model indicates that other factors likely explain from 70 to > 99% of variation in vagrancy, depending on the species. Additionally, the combination of geomagnetic disturbance and other barriers to accurate navigation, like cloud cover or dense fog, may interact to drive vagrancy. In some migratory taxa not examined here—such as seabirds—alternative factors like storms or exploratory wandering may be even more important^58–60^. Other data sources that include records of species not routinely banded, like rare bird lists and citizen science occurrence records^61^, may be particularly useful for analyzing vagrancy rates among these species. Unlike other purported drivers of vagrancy like population size and weather, geomagnetic disturbance and solar activity are extensively and accurately monitored and follow regular cycles, potentially lending some predictability to the largely stochastic phenomenon of avian vagrancy.

Periodic and variable vagrancy due to misorientation and other mechanisms has potentially wide-ranging implications for avian populations, both negative and positive. With North American avian populations broadly in decline^62^, vagrancy may exacerbate these trends by increasing mortality rates during the already-dangerous migratory season^63,64^. Conversely, vagrancy may facilitate range expansions under climate change by seeding individuals into newly climatically suitable areas^65^.

The perturbation of normal migratory patterns also likely has wider-ranging ecological ramifications. Avian migrants are responsible for the widespread and rapid dispersal of parasites, pathogens, and propagules—linking distinct ecosystems across wide geographical areas^7,8,66–68^. With the speed and probability of range expansions heavily impacted by rare, long-distance dispersal events^1^, vagrant birds transporting organisms far beyond their expected range have an outsized impact on the range expansion of these populations^8^. Understanding the causes of avian vagrancy is critical to understanding the threats to, and resilience of, avian populations, and predicting the rare ecological events that shape species’ ranges in a changing world.

## Methods

To investigate whether vagrancy is associated with geomagnetic disturbance and solar activity, we developed a method for quantifying the relative vagrancy of spatiotemporal records for 152 North American landbird species (n_fall_ = 150, n_spring_ = 124). While vagrancy is often treated as a binary classification (i.e., an individual is either a vagrant or not) and then used as a discrete variable (i.e., a count of total vagrants in an area)^16,18^, here we calculated it as a continuous variable by combining two large-scale ornithological datasets—captures and encounters of individually marked birds from the USGS Bird Banding Lab (BBL)^49^ and weekly, species-specific abundance maps for the continental United States from the eBird Status and Trends (hereafter, eBird S&T; via the R package ‘ebirdst’, version 2.1.0)^69^. Banding records have the advantage over other potential databases of vagrancy records (such as eBird or rare bird lists) in that efforts are long-term, continent-wide, have limited false positives, and have only one record per individual. Additionally, eBird S&T has the advantage over static range maps in that they provide weekly predictions and incorporate relative abundance. With these two data sources, we constructed a species-specific vagrancy value (Fig. S1), that measures the spatiotemporal rarity for every banding record. Inclusion of all banding records rather than just rare records allowed for the analysis of the dispersion of whole species populations, mitigating the potential bias of effort in banding operations (i.e., more vagrant records with greater effort). We then used hierarchical Bayesian random-effects models to estimate the strength of the association between geomagnetic disturbance, solar activity, and avian vagrancy.

### Species data and inclusion

We considered all full—or partial-migrant landbird species with a breeding, non-breeding, or migratory range in the United States or Canada. To do this, we used species distribution maps accessed through Birds of the World^70^. Landbird species likely to be caught through banding efforts (excluding species like raptors, nightjars, and swifts) that regularly occur in > 3 but < 45 of the 48 continental U.S. states were included in the analysis. This step was taken to eliminate widespread species and those with only a small breeding range (e.g., only in the southernmost U.S.), thereby only including species that have the possibility of being banded outside of their ‘normal’ ranges within the U.S.

Banding records from 1960 to 2019 were provided by the BBL. Only first-time banding records and subsequent visual encounters were included in the analysis—recaptures were excluded as they are inconsistently reported (Bystrak, pers. comm. 2020). Encounters, which are reported opportunistically by either bird banders or members of the public, made up a small percentage (0.5%) of all data used in the analysis. We included all captures of wild birds not involved in manipulative experiments (banding code = 3) and excluded all records where precision of banding locations was > 10 km. Each banding record included the date, latitude and longitude (and precision), species, and age (if known;^71^). Banding records were filtered to those captures that occurred during the species-specific migration period as defined by eBird S&T^69^. eBird S&T approximates stationary and migratory periods by determining when the distribution of whole species population is moving^69^. Our use of banding records within species-specific eBird S&T migratory periods was designed to maximize the proportion of migrant birds in the analysis, but likely excludes some early and late records of migrating individuals.

Banding records of species that underwent taxonomic divisions or aggregations during the study period were eliminated if the date occurred during a period in which the species identity according to modern taxonomy is indeterminate (see Supplement 2). Taxonomic reclassifications were not considered when species divisions/aggregations would only affect records from outside North America, such as the split of a Southern American taxon, Chestnut-collared Swallow (*Petrochelidon rufocollaris*) from its North American counterpart, Cave Swallow (*Petrochelidon fulva*). In these cases, we assumed all banding records during the study period were of the North American species. For a full list of periods where species records were excluded, see Supplement 2. Species with < 100 total records during either fall or spring migration were excluded from analyses for that season. Filtering narrowed our analysis to 152 species, of which 150 were included in the fall analysis, and 124 in the spring analysis. Of the 152 species, 135 were Passeriformes, 9 were Caprimulgiformes, 5 were Piciformes, 2 were Columbiformes, and 1 Cuculiformes (full species list in Supplement 2).

### eBird predicted abundance maps

High-resolution weekly relative abundance maps for each of the 152 species were downloaded from eBird S&T (via the R package ebirdst, version 0.2.1). These maps—which predict the relative abundance of species across space and through time—are created using a combination of statistical and machine learning models that integrate land-cover data and millions of citizen-science bird checklists while accounting for potential biases in sampling^72^. eBird S&T maps were used both to estimate the average migratory length and breeding latitude of each species, as well as to calculate the vagrancy index for each banding record (Fig. S1).

### Geomagnetic and solar activity data

The daily American relative sunspot number from 1959 to 2019 was downloaded from the LISIRD service of the Laboratory of Atmospheric and Space Physics at the University of Colorado, Boulder (http://lasp.colorado.edu/lisird). We chose American relative sunspot number as a proxy for broadband solar radiofrequency noise rather than using a measure of a single frequency because of the impact of a range of frequencies on biological sensors^31,73^. The daily global Ap index, a metric of disturbance to the Earth’s magnetic field, was downloaded from the International Service for Geomagnetic Indices (‘Kp’ dataset; http://isgi.unistra.fr/data_download.php) for the same period. For each of these indices, a rolling average of the previous 21 days was calculated for each day during the study period (1960–2019). We used 21 days to account for the latency between when the effect occurs and the day in which the affected individual was likely to be captured. A time period that was too short would lead us to potentially miss an influential geomagnetic disturbance or solar storm, while a period that was too long would dampen the effect of an acute geomagnetic disturbance or solar storm.

### Vagrancy index

We calculated the spatiotemporal rarity for each banding record by comparing the banding location to the weekly species-specific abundance probability maps from eBird. Our vagrancy index was developed to measure and compare the rates of vagrancy across years within species. For each species and week, 10,000 random spatial points were simulated proportional to their relative abundance probability as given by the eBird S&T maps (Fig. S1A). Using these points unique to each species and week of the year, we conducted a mean nearest-neighbor analysis (k = 10) for each banding record of a given species and week (Fig. S1B), such that the raw vagrancy index was equal to the mean distance (km) from the 10 nearest eBird-derived points (Fig. S1C). Banding points that fell outside of the modeled range of eBird S&T maps (i.e., at extreme latitudes, or where eBird coverage is otherwise poor) were excluded. The distributions of vagrancy index values differ across species due to sampling differences in the banding data (Figs. 1, 3) and are only comparable within species. We account for the species-specific nature of these values in our statistical analysis by modeling species-specific gamma distributions (see “Hierarchical models of vagrancy” section).

### Migration length, breeding latitude, and nocturnal versus diurnal designation

For each species, we calculated migration length and breeding latitude using eBird S&T. For each week within both the eBird-defined breeding and non-breeding seasons, 10,000 random points were drawn from the relative abundance species distribution maps. We calculated the centroid of these points for each season and used the Haversine distance between breeding and non-breeding centroids as a measure of migration length^74^. For species with extensive non-breeding distributions in South America that are not covered by eBird map products (n = 5 species, see Supplement 2), we manually estimated breeding and non-breeding centroids and migration length values of the North American population by consulting population-specific information and distribution maps available on Birds of the World^70^ and eBird^69^.

We designated species as exhibiting either diurnal, nocturnal, both, or unknown migration strategies based on species-specific information from “Birds of the World”^70^. Species with evidence of migrating during either the day or night were considered diurnal or nocturnal, respectively. Migrants known to make low-altitude dawn flights or reorientation flights were not considered diurnal. In addition to using species-specific information, we inferred migratory timing strategies for certain taxonomic groups with evidence of only a single strategy. Thus, all members of the Parulidae family were classified as nocturnal, while members of the Trochilidae, Hirundinidae, and Fringillidae families were all classified as diurnal.

### Hierarchical models of vagrancy

We used hierarchical models in a Bayesian context to investigate whether vagrancy in the fall or spring migration seasons is associated with geomagnetic disturbance and/or solar activity. This flexible Bayesian generalized linear mixed model approach allowed us to model the substantial heterogeneity in our data (e.g., variation among species and across years), modeling species-specific and group-level effects through the use of random intercepts and random slopes. The Bayesian approach allowed us to quantify our uncertainty in parameter estimates and effectively model missed data by treating parameter estimate probabilistically^75^.

In all analyses, we modeled vagrancy as a gamma-distributed variable with a shape parameter that varied by species. The gamma distribution is used here as it provides a flexible way to model continuous positive values^76^, a condition that is met with our response variable (vagrancy index). We assessed the ability of the gamma distribution to fit the data for each model through posterior predictive checks (see “Model conditions and checks” section). Due to differences in the species included and the relative lack of age information in the spring dataset, we used structurally identical but independent models for each season. We fit four models to test for the effects of geomagnetic disturbance and solar activity during both the fall and spring migration season: two for the fall season and two for the spring season (“Models 1–4: effect of geomagnetic disturbance or solar activity on vagrancy in fall or spring”, Eqs. 1–3). Fall models utilized a thinned dataset (n = 1,331,471) to prioritize records from low-density years and species. The number of banding records included for each species was limited to 20,000. For species with more than 20,000 records, the probability for inclusion of each record was inversely proportional to the number of all records from that species in that year, such that records from years that had less data were more likely to be sampled. Thinning was necessary due to computational limitations—runtimes with the full fall dataset (n =  ~ 3 million records) were projected to exceed 90 days using high performance computing resources.

In order to include bird records with unknown age (a common feature of bird banding data), our fall models used Bayesian imputation^77^ to estimate unknown age data (See Supplement 2 for model code). This approach considers the age of an individual with missing information to be probabilistic, allowing for the use of all records, regardless of the presence of age information. Because of the large number of banding records with unknown age (68%) in the spring dataset, the models using Bayesian imputation did not converge due to identifiability issues of the age-related parameters. As such, we excluded records where age was unknown and fit the spring model with a dataset filtered to include only species with more than 100 records of known age (124 species; n = 931,121). We chose this approach rather than fitting a structurally different model with age parameters excluded to be able to directly compare the output of the two models. Species in each dataset have representatives from the same 19 avian families, have similar migration lengths, measured as the distance between the centroids of breeding and non-breeding ranges (mean, fall = 2251 km, spring = 2330 km) and breeding latitudes (mean, fall = 42.29°N, spring = 42.44°N). For our model investigating the interaction between geomagnetic disturbance and solar activity (Models 5–6, Eqs. 4–5), we utilized a thinned version of the full fall and spring dataset (n_fall_ = 1,331,471, n_spring_ = 1,104,141), including records with all age-classes represented, including with unknown age. In this analysis, we were able to use all records regardless of age because these models excluded age-specific terms (see “Models 5–6: interactive effect of geomagnetic disturbance and solar activity on vagrancy in spring or fall” section). For all models, we normalized migration length, breeding latitude, and indices of geomagnetic disturbance and solar activity to have a mean of zero and standard deviation of 1.

#### Models 1–4: effect of geomagnetic disturbance or solar activity on vagrancy in fall or spring

To estimate the association between vagrancy and geomagnetic disturbance/solar activity, and to the degree to which migratory length, breeding latitude, and age are associated with the strength of this relationship, we constructed a Bayesian hierarchical model with the following structure:

1 $$\begin{aligned} y_{j} \sim gamma\left( {shp_{i} ,\frac{{shp_{i} }}{{e^{{lp_{j} }} }}} \right) \\ lp_{j} = \alpha_{t,i} + \beta_{t,i} *X_{j} + \lambda_{i} *age_{j} + \nu_{i} *age_{j} *X_{j} \\ \end{aligned}$$

where the vagrancy index, *y,* for each record, *j*, is modeled as a gamma-distributed random variable, and where *t* represents the year, *i* represents the species, and *lp* is the linear predictor representing the model-predicted vagrancy for record *j*. Parameter $\alpha_{t,i}$ is the intercept, $\beta_{t,i}$ is the effect of geomagnetic disturbance or solar activity on vagrancy, $X$ is the 21-day rolling average of geomagnetic disturbance (models 1 and 2) or solar activity (models 3 and 4), $\lambda_{i}$ is the effect of age on vagrancy, and $\nu_{i}$ is the effect of age on sensitivity to geomagnetic disturbance or solar activity. The *age* term is a binary indicator, with 0 representing individuals older than one year (‘after-hatch-year’ and equivalent for fall data, ‘after-second-year’ and equivalent for spring data), and 1 representing individuals younger than one year (‘hatch-year’ for fall data, ‘second-year’ for spring data). Fall models used Bayesian imputation^78^ to estimate unknown age data.

We modeled species-specific parameters hierarchically with:

2 $$\begin{aligned} shp_{i} \sim & N\left( {\mu_{shp} ,\sigma_{shp} } \right) \\ \alpha_{t,i} \sim & N\left( {\gamma_{i} ,\sigma_{\alpha } } \right) \\ \beta_{t,i} \sim & N\left( {\mu_{{\beta_{i} }} ,\sigma_{\beta } } \right) \\ \lambda_{i} \sim & N\left( {\mu_{\lambda } ,\sigma_{\lambda } } \right) \\ \nu_{i} \sim & N\left( {\mu_{\nu } ,\sigma_{\nu } } \right) \\ \end{aligned}$$

where *shp* is the shape parameter of the gamma distribution, $\gamma_{i}$ is the mean vagrancy of a species across all years, $\mu_{{\beta_{i} }}$ represents the species-specific sensitivity to geomagnetic disturbance or solar activity, and $\mu_{\lambda }$ and $\mu_{\nu }$ represent the cross-species impact of age on vagrancy and the cross-species impact of age on sensitivity to geomagnetic disturbance or solar activity, respectively. The $\sigma$ terms here and below represent the standard deviation of each parameter.

Given that we were interested in the influence of species-specific traits on vagrancy rates, we modeled the effect of geomagnetic disturbance/solar activity as a function of species-specific traits:

3 $$\begin{aligned} \gamma_{i} \sim N\left( {\mu_{\gamma } ,\sigma_{ \gamma } } \right) \\ \mu_{\beta i} \sim N\left( {\delta_{i} ,\sigma_{ \delta } } \right) \\ \delta_{i} = \omega + \psi *migration\,length_{i} + \eta *breeding\,latitude_{i} \\ \end{aligned}$$

where $\mu_{\gamma }$ is the mean vagrancy across all species, $\omega$ represents the cross-species mean impact of geomagnetic disturbance or solar activity on vagrancy at mean migration length and breeding latitude, $\psi$ represents the effect of migration length on sensitivity, and $\eta$ represents the effect of breeding latitude on sensitivity.

#### Models 5–6: interactive effect of geomagnetic disturbance and solar activity on vagrancy in spring or fall

To investigate the potential interaction between geomagnetic disturbance and solar activity in the fall (model 5) and spring (model 6), we used a simplified model that included both geomagnetic disturbance and solar activity (and their interaction), but removed age-related terms to facilitate model tractability:

4 $$\begin{aligned} y_{j} \sim gamma\left( {shp_{i} ,\frac{{shp_{i} }}{{e^{{lp_{j} }} }}} \right) \\ lp_{j} = \alpha_{t,i} + \beta_{t,i} *X_{{1_{j} }} + \theta_{i} *X_{{2_{j} }} + \omega_{i} *X_{{1_{j} }} *X_{{2_{j} }} \\ \end{aligned}$$

where the vagrancy index, *y*, for each record*, j*, is again modeled as a gamma-distributed random variable, and where *t* represents the year, *i* represents the species and *lp* is the linear predictor representing the model-predicted vagrancy for record *j*. Parameter $\alpha_{t,i}$ is the intercept, $\beta_{i}$ is the effect of geomagnetic disturbance on vagrancy, $\theta_{i}$ is the effect of solar activity on vagrancy, and $\omega_{i}$ is the interaction term between geomagnetic disturbance and solar activity. X_1_ is the 21-day rolling average of geomagnetic disturbance, and X_2_ is the 21-day rolling average of the sunspot number.

Parameters were modeled hierarchically, where:

5 $$\begin{aligned} shp_{i} \sim & N\left( {\mu_{shp} ,\sigma_{shp} } \right) \\ \alpha_{t,i} \sim & N\left( {\mu_{\alpha_{i}} ,\sigma_{\alpha } } \right) \\ \beta_{i} \sim & N\left( {\mu_{\beta } ,\sigma_{\beta } } \right) \\ \theta_{i} \sim & N\left( {\mu_{\theta } ,\sigma_{\theta } } \right) \\ \omega_{i} \sim & N\left( {\mu_{\omega } ,\sigma_{\omega } } \right) \\ \mu_{\alpha_{i}} \sim & N\left( {\gamma ,\sigma_{{\mu_{\alpha } }} } \right) \\ \end{aligned}$$

with $\mu_{\beta }$ representing the cross-species effect of geomagnetic disturbance on vagrancy, $\mu_{\theta }$ representing the cross-species effect of solar activity on vagrancy, $\mu_{\omega }$ representing the cross-species interaction effect between the two, and $\sigma$ terms representing the process variance. We estimated the proportion of total variance explained by the covariates (Bayesian R^2^) using an method adapted for analyzing Bayesian models^79^. For each species, we calculated the variance of the predicted vagrancy index ($g_{rep}$) and the residual, unexplained variance ($\in$),

6 $$\begin{aligned} g_{{rep_{j} }} = \beta_{t,i} *X_{{1_{j} }} + \theta_{i} *X_{{2_{j} }} + \omega_{i} *X_{{1_{j} }} *X_{{2_{j} }} \\ \in_{j} = y_{j} - g_{{rep_{j} }} \\ \end{aligned}$$

We then calculated the percent variance explained for a given species as the proportion of the total variance explained by the covariates,

7 $$\frac{{V_{{g_{rep} }} }}{{V_{{g_{rep} }} + V_{ \in } }}$$

This produced a posterior distribution of percent variance explained, from which we calculated the median for each species. We report the cross-species estimate as the median of the species-level estimates.

### Model conditions and checks

All models were fit using the rstan package (version 2.21.2)^80^ to interface with stan^81^ using R version 4.1 and summarized using MCMCvis (version 0.15.3)^82^. Models were run using the UCLA Hoffman2 Cluster using parallelization with each chain of the Bayesian models being run on a separate core. Details of the model runs with convergence diagnostics and posterior predictive checks are provided in Supplement 2.

### Phylogenetic post-hoc test

Due to identifiability issues when included in the model, phylogenetic relationships were not directly accounted for in this modeling framework. As such, we used a post-hoc bootstrapping approach to determine the possible effect that phylogeny might have the on sensitivity of vagrancy to geomagnetic disturbance and solar activity. We calculated Blomberg’s K^83^ for species-level sensitivities estimated by each model using the R package picante^84^ and 100 phylogenetic tree subsets^85^ from birdtree.org. Our analysis suggests there is no phylogenetic signal in any of the six models fits (K < 0.1, much lower than 1, the expectation under Brownian motion, Supplement 2).

## Supplementary Information

Supplementary Figures.

Supplementary Information.

## Data Availability

All code to process the data, conduct the analysis and create the figures in the manuscript are provided as Supplement 2. Species-specific data information, including number of records, migration length and breeding latitude are included in Supplement 2. The datasets analyzed in during the current study are available in the ScienceBase repository, (https://www.sciencebase.gov/catalog/item/60914db3d34e791692e13a22).
