# Supplementary material for: Geomagnetic disturbance associated with increased vagrancy in migratory landbirds
Source: Sci Rep. 2023 Jan 9;13:414. doi: 10.1038/s41598-022-26586-0 (PMC9829733; doi:10.1038/s41598-022-26586-0)
Supplement: Supplementary file 1 — Supplementary Figures. [file 41598_2022_26586_MOESM1_ESM.docx]

**SUPPLEMENT 1**


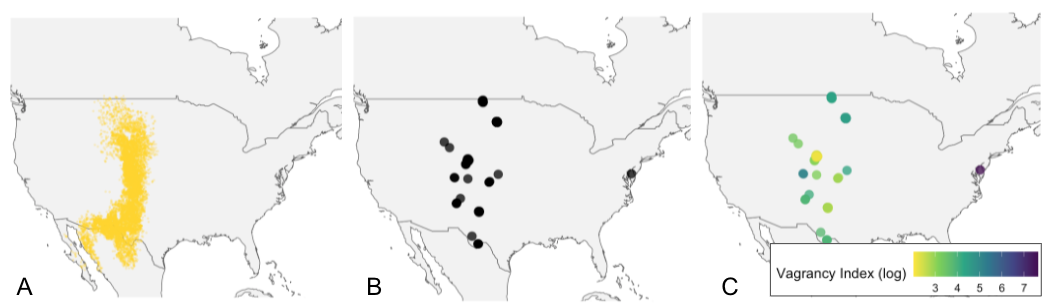


**Figure S1.** Vagrancy for every banding record was calculated as a measure of spatio-temporal rarity. (A) 10,000 points were randomly drawn from predicted weekly relative abundance maps proportional to their relative abundance probability in order to calculate the expected spatio-temporal abundance for a species during a given week. (B) The expected range was compared to actual banding records for a species in that week. (C) This comparison resulted in a vagrancy index for every record, derived as the mean of the 10 nearest-neighbor distances between observed banding records (B) and the expected distribution (A). Maps show vagrancy calculation steps for the week of August 17-23 for the Lark Bunting. Vagrancy values – plotted on a log scale – represent the relative spatiotemporal rarity of each record, rather than a using a strict binary cutoff (i.e., vagrant or not). Vagrancy scores for all Lark Bunting records during the fall migration season are shown in Fig. 1. Maps were created using the packages rnaturalearth (v. 0.1.0, https://github.com/ropensci/rnaturalearth) and ggplot2 (v. 3.3.5, https://ggplot2.tidyverse.org).


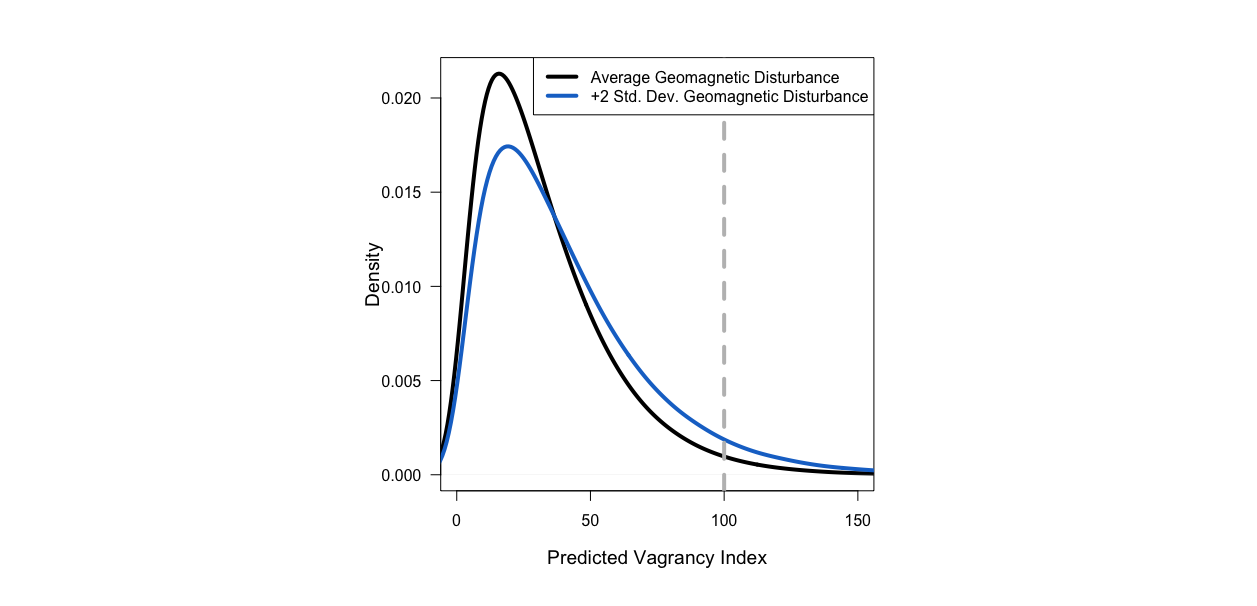


**Figure S2.** Predicted distribution of vagrancy index values at average and high (+2 standard deviations) geomagnetic disturbance for the typical (average) species during the fall migration season using results from Model 1. Geomagnetic disturbance increases the dispersion of populations, lengthening the tails of the distribution of spatiotemporal rarity.


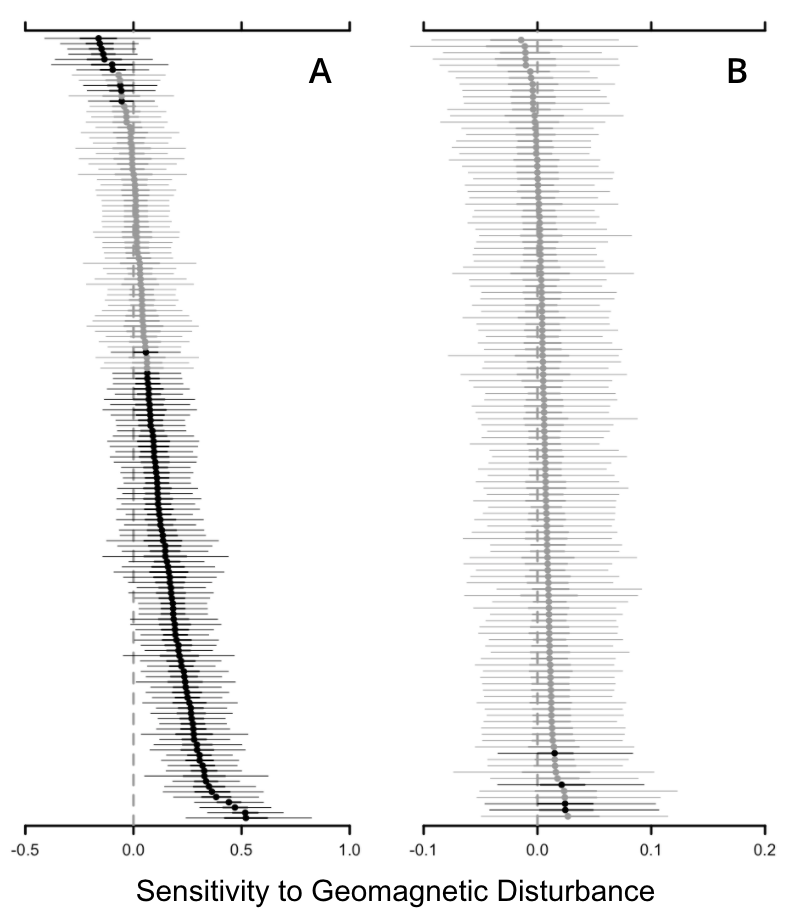


**Figure S3.** Parameter estimates for species-level sensitivity to geomagnetic disturbance in the fall (A) and spring (B) migration seasons ($\mu_{\beta_{i,}}$, Eqn. 2,3). Posterior medians are indicated by circles, 50% CrI by the thick lines, and 95% CrI by the thin lines. Black circles indicate species-specific estimates where the CrI does not include 0. Order of species is determined by median estimate and is not consistent between the two figures.


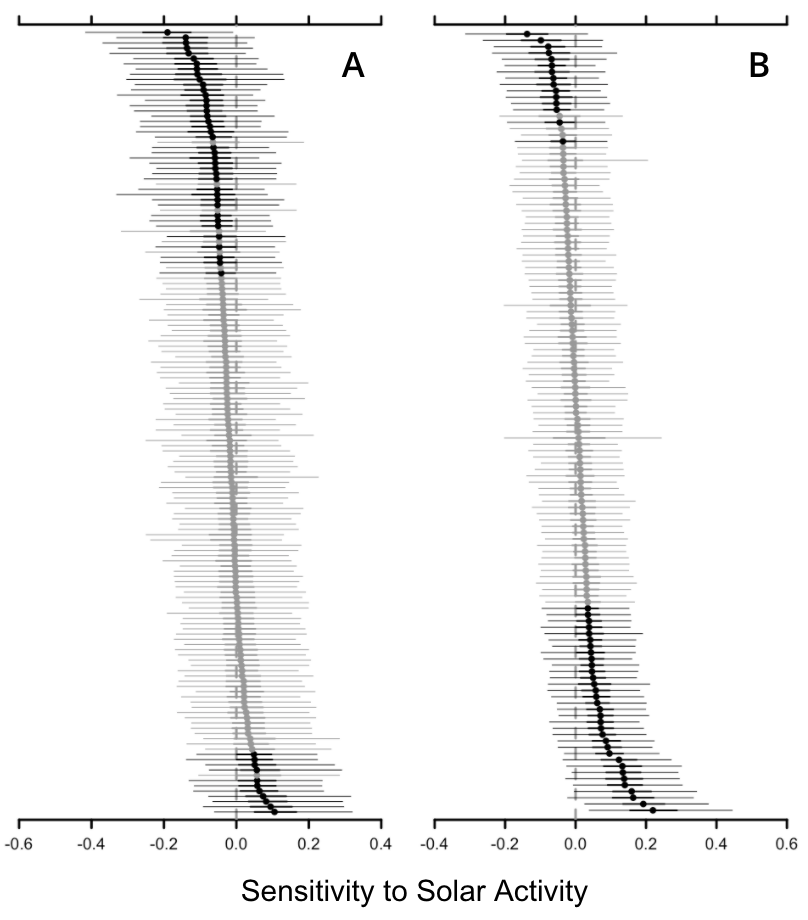


**Figure S4.** Parameter estimates for species-level sensitivity to solar activity in the fall (A) and spring (B) migration seasons ($\mu_{\beta_{i,}}$, Eqn. 2,3). Posterior medians are indicated by circles, 50% CrI by thick lines, and 95% CrI by thin lines. Black circles indicate species-specific estimates where the CrI does not include 0. Order of species is determined by median estimate and is not consistent between the two figures.


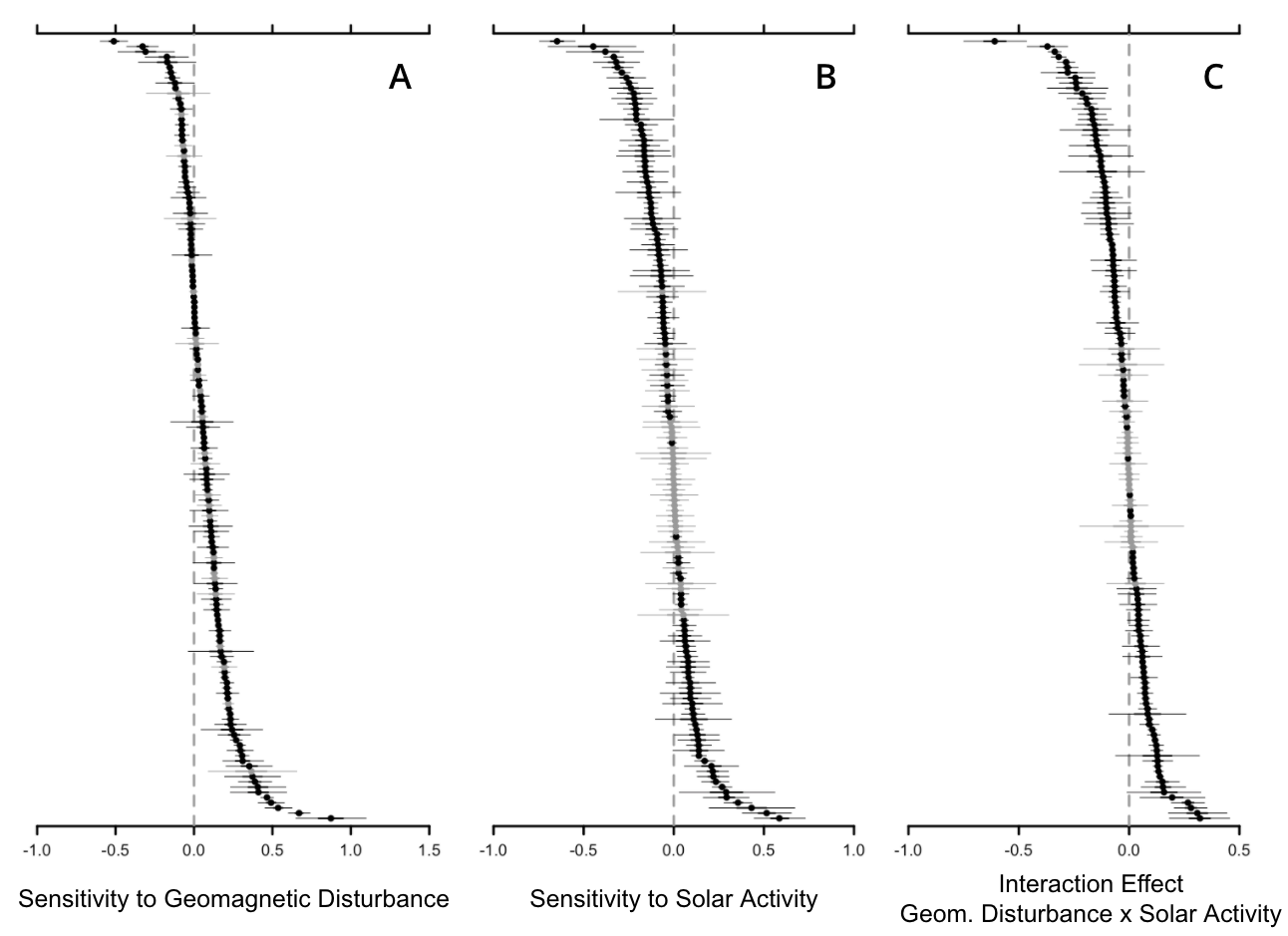


**Figure S5.** Parameter estimates for species-level sensitivity to geomagnetic disturbance (A, $\beta_{i},$ Eqn. 5), solar activity (B, $\theta_{i},$ Eqn. 5) and the interaction between the two (C, $\omega_{i},$ Eqn. 5) during the fall migration season. Posterior medians are indicated by circles, 50% CrI by the thick lines, and 95% CrI by the thin lines. Black circles indicate species-specific estimates where the CrI does not include 0. Order of species is determined by median estimate and is not consistent between the three figures.


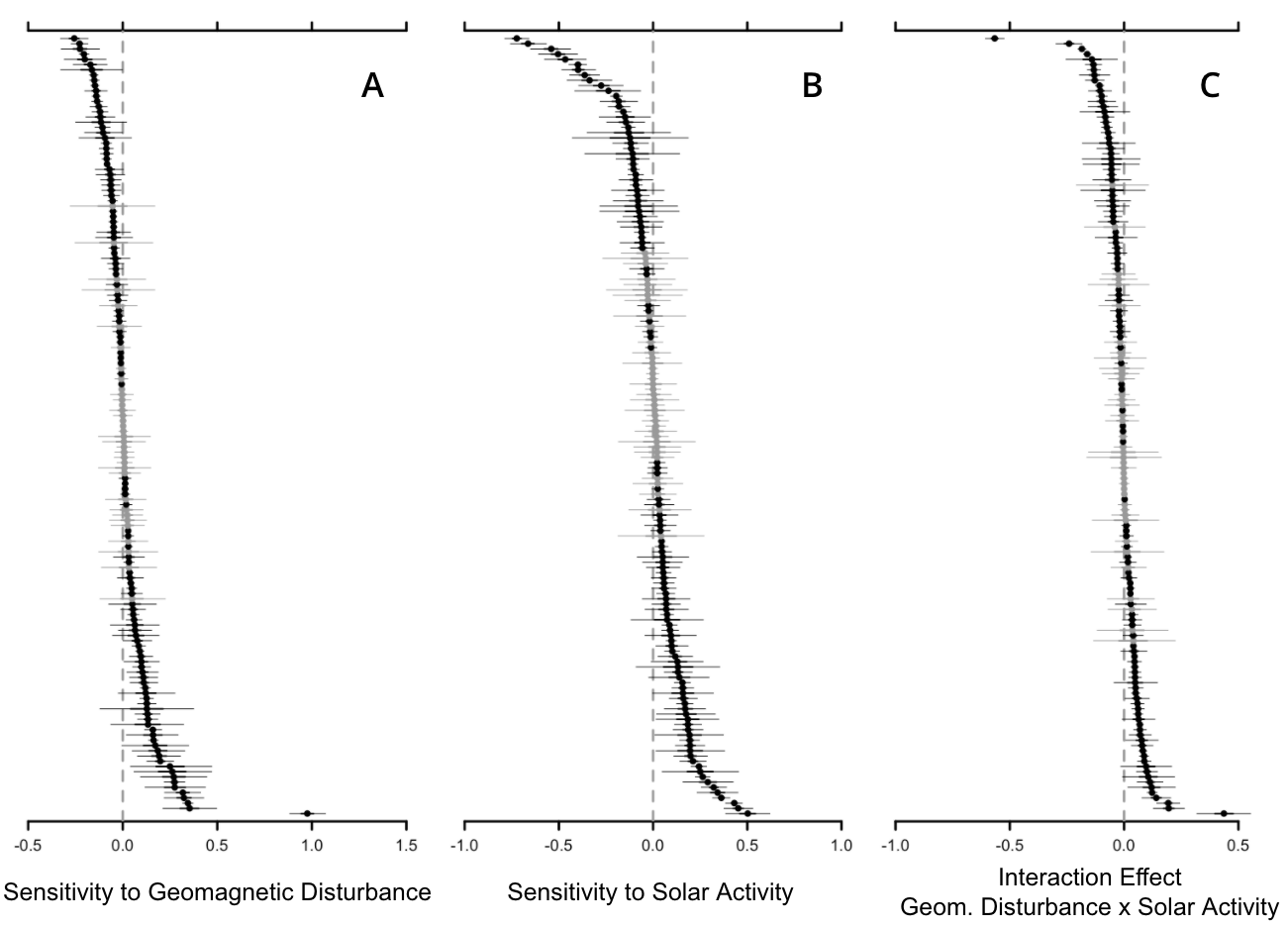


**Figure S6.** Parameter estimates for species-level sensitivity to geomagnetic disturbance (A, $\beta_{i},$ Eqn. 5), solar activity (B, $\theta_{i},$ Eqn. 5) and the interaction between the two (C, $\omega_{i},$ Eqn. 5) during the spring migration season. Posterior medians are indicated by circles, 50% CrI by the thick lines, and 95% CrI by the thin lines. Black circles indicate species-specific estimates where the CrI does not include 0. Order of species is determined by median estimate and is not consistent between the three figures.


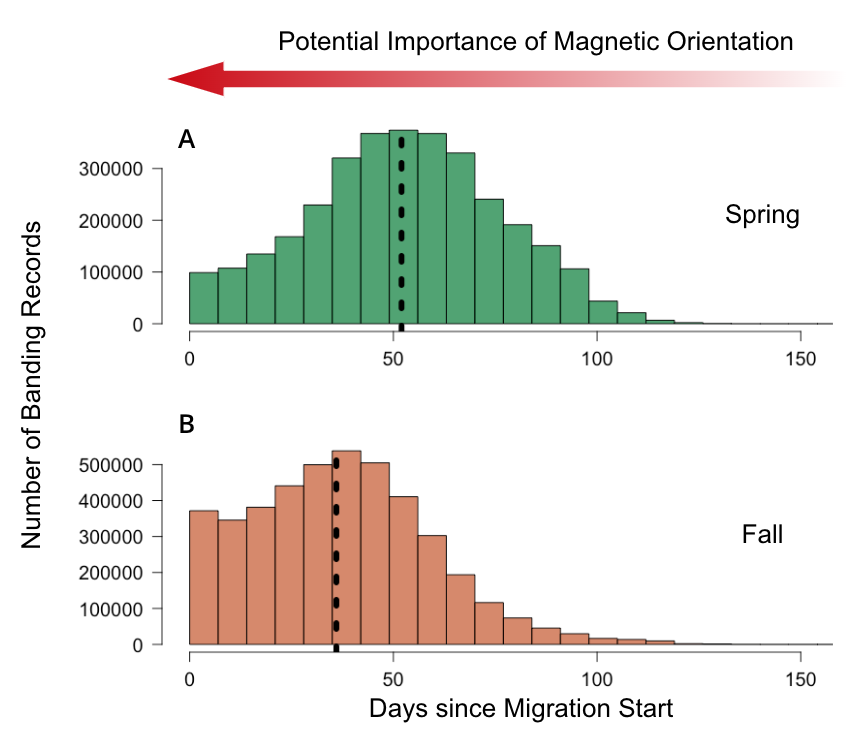


**Figure S7.** Distribution of all banding records included in the analysis for the (A) spring and (B) fall migration season. Records are plotted by date of capture since the start of species-specific migratory periods, as defined by eBird S&T. Medians of each distribution are represented with black lines. Birds in the spring were captured relative to the initiation of migration than birds in the fall (difference in median capture = 16 days, 99% CI using bootstrapping = [16,16], p < .01). The hypothesized importance of magnetic information for navigation is indicated with the red arrow, as species farther from their migratory destination are expected to rely more on magnetic orientation (Mouritsen, 2018). With spring banding records skewed away from the onset of migration, detecting the effect of geomagnetic disturbance or solar activity on vagrancy is less likely.


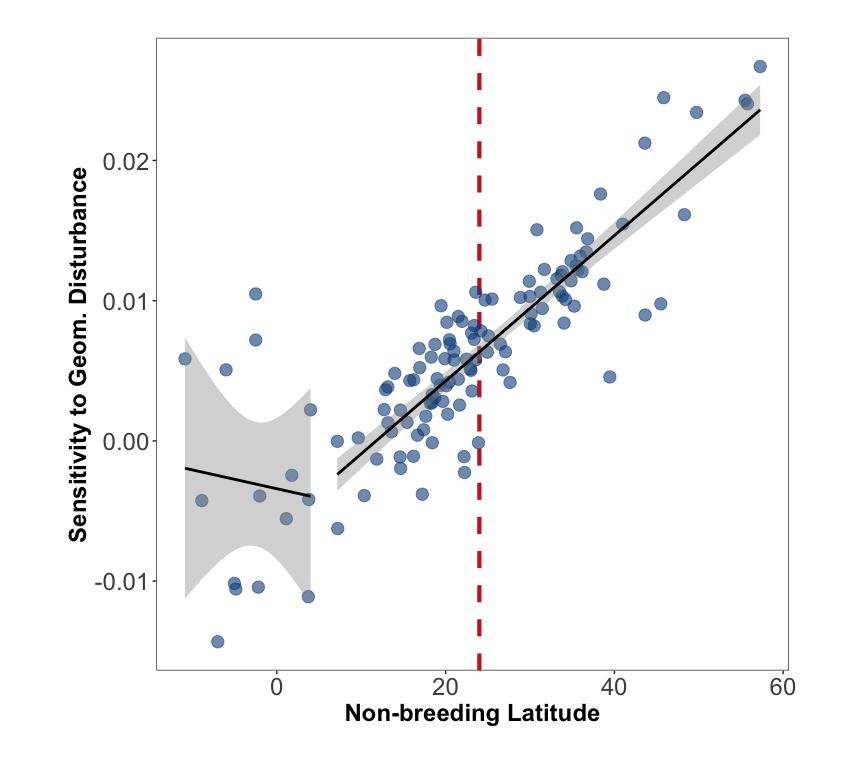


**Figure S8.** Posterior median estimates of species-level sensitivity to geomagnetic disturbance during spring migration and average latitude of non-breeding ranges (°N) of each species (blue dots). Note that posterior medians are informed by breeding latitude and migratory length (see Equations 1–3). Here, we derived non-breeding range estimates from eBird Science and Trends maps, except in 5 cases where data was limited (see methods for details). We fit a segmented regression (black lines, 95% CI as grey envelope) with a model-estimated breakpoint using the R package SiZer (v. 0.1.7). The lack, and relative weakness, of the association between geomagnetic disturbance and vagrancy among species with southernly non-breeding ranges supports our hypothesis that poor data coverage south of the United States (left of red dotted line) masks the role of geomagnetic disturbance by limiting the data collected during the early migratory period in which species are more likely to rely on magnetic orientation.


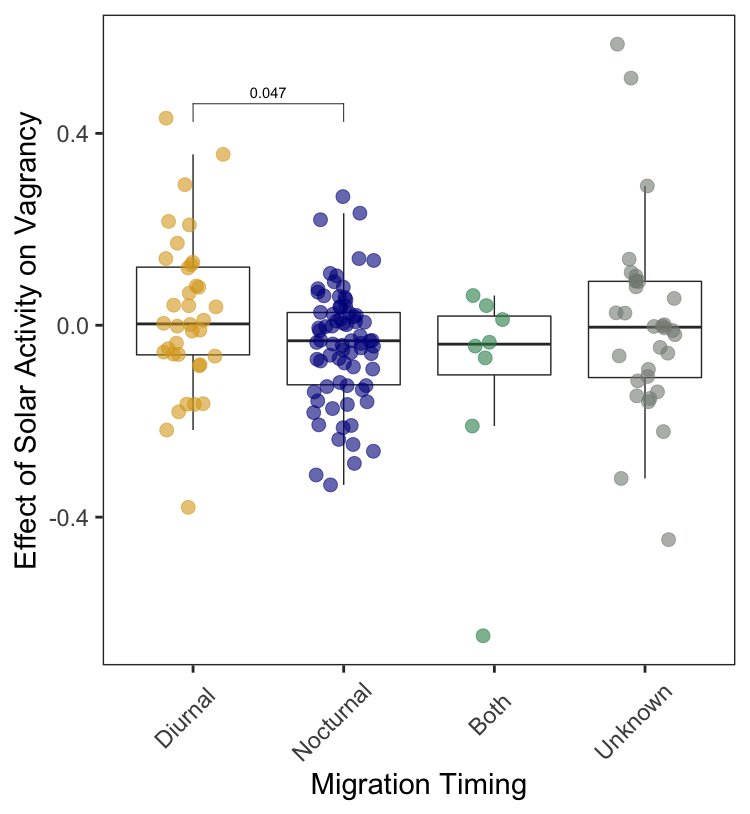


**Figure S9.** Posterior median estimates for species-level sensitivity to solar activity grouped by the time of day in which species are known to migrate. We found that diurnal migrants are the most sensitive to solar activity (t-test, diff. between mean sensitivity${(\theta}_{i_{fall}})$ in nocturnal, diurnal groups = 0.062, t = -2.03, p = 0.047).
