# Supplementary material for: Geomagnetic disturbance associated with increased vagrancy in migratory landbirds
Source: Sci Rep. 2023 Jan 9;13:414. doi: 10.1038/s41598-022-26586-0 (PMC9829733; doi:10.1038/s41598-022-26586-0)
Supplement: Supplementary file 2 — Supplementary Information. [file 41598_2022_26586_MOESM2_ESM.docx]

**SUPPLEMENT 2**

All code to conduct the analysis is available via: <https://github.com/bentonelli/Bird_Vagrancy_Geomagnetic_Disturbance>

All banding data is available via ScienceBase: <https://www.sciencebase.gov/catalog/item/613f7134d34e1449c5d35c2c>
